# Magnetic Nanoparticles and Magnetic Field Exposure Enhances Chondrogenesis of Human Adipose Derived Mesenchymal Stem Cells But Not of Wharton Jelly Mesenchymal Stem Cells

**DOI:** 10.3389/fbioe.2021.737132

**Published:** 2021-10-18

**Authors:** Luminita Labusca, Dumitru-Daniel Herea, Anca Emanuela Minuti, Cristina Stavila, Camelia Danceanu, Petru Plamadeala, Horia Chiriac, Nicoleta Lupu

**Affiliations:** ^1^ National Institute of Research and Development for Technical Physics, Iasi, Romania; ^2^ Orthopedics and Traumatology Clinic County Emergency Hospital Saint Spiridon, Iasi, Romania; ^3^ Faculty of Physics, Alexandru Ioan Cuza University, Iasi, Romania; ^4^ Pathology Department County Children Emergency Hospital Saint Mary, Iasi, Romania

**Keywords:** adipose derived stem cells, wharton jelly mesenchymal stem cells, magnetic nanoparticles, magnetic field, chondrogenesis, adipose derived mesenchymal stem cell

## Abstract

**Purpose:** Iron oxide based magnetic nanoparticles (MNP) are versatile tools in biology and medicine. Adipose derived mesenchymal stem cells (ADSC) and Wharton Jelly mesenchymal stem cells (WJMSC) are currently tested in different strategies for regenerative regenerative medicine (RM) purposes. Their superiority compared to other mesenchymal stem cell consists in larger availability, and superior proliferative and differentiation potential. Magnetic field (MF) exposure of MNP-loaded ADSC has been proposed as a method to deliver mechanical stimulation for increasing conversion to musculoskeletal lineages. In this study, we investigated comparatively chondrogenic conversion of ADSC-MNP and WJMSC with or without MF exposure in order to identify the most appropriate cell source and differentiation protocol for future cartilage engineering strategies.

**Methods:** Human primary ADSC and WJMSC from various donors were loaded with proprietary uncoated MNP. The *in vitro* effect on proliferation and cellular senescence (beta galactosidase assay) in long term culture was assessed. *In vitro* chondrogenic differentiation in pellet culture system, with or without MF exposure, was assessed using pellet histology (Safranin O staining) as well as quantitative evaluation of glycosaminoglycan (GAG) deposition per cell.

**Results:** ADSC-MNP complexes displayed superior proliferative capability and decreased senescence after long term (28 days) culture *in vitro* compared to non-loaded ADSC and to WJMSC-MNP. Significant increase in chondrogenesis conversion in terms of GAG/cell ratio could be observed in ADSC-MNP. MF exposure increased glycosaminoglycan deposition in MNP-loaded ADSC, but not in WJMSC.

**Conclusion:** ADSC-MNP display decreased cellular senescence and superior chondrogenic capability *in vitro* compared to non-loaded cells as well as to WJMSC-MNP. MF exposure further increases ADSC-MNP chondrogenesis in ADSC, but not in WJMSC. Loading ADSC with MNP can derive a successful procedure for obtaining improved chondrogenesis in ADSC. Further *in vivo* studies are needed to confirm the utility of ADSC-MNP complexes for cartilage engineering.

## Introduction

Musculoskeletal diseases are an increasing burden worldwide and a principal cause of persistent pain and disability. Among them, the incidence of degenerative joint diseases (DJD), mainly of osteochondral defects (OD) and osteoarthritis (OA) is on the rise, affecting hundreds of millions of people worldwide (Kloppenburg and Berenbaum, 2020). Ageing population, sedentary life style but as well increased occurrence of traumatic injuries due to traffic accidents, war casualties or natural disasters have been implicated in DJD. Their impact is even more dramatic due to the fact that such lesions do not heal and have as consequences permanent disabilities and reduced quality of life. In addition, DJD is getting “younger”, 16–17% of adults below 40 and up to 60% of adults of 40–65 years of age are reported to having various forms of the disease (Merkely et al., 2018). Current therapies are mainly symptomatic, failing to offer effective joint anatomical and functional rehabilitation. Total joint replacement still represents the ultimate method for treating DJD-induced joint deterioration. Regenerative therapies promise to deliver biological restauration of joint surface and to offer permanent and durable anatomical and functional recovery. Despite more than 30 years of basic and translational research in the field, current available products for joint resurfacing are failing to deliver consistent disease modifying therapeutic solutions. The most appropriate cell source for cartilage bioengineering is still under debate. ADSC are extensively investigated for their use as cell sources in regenerative medicine (RM) (Zizhen et al., 2019; Lima et al., 2015). ADSC are relatively easy to extract by means of enzymatic or mechanical methods from the adipose tissue derived from elective cosmetic procedures. ADSC are available as both autologous and allogeneic sources, can derive larger number of elements per tissue volume (Si et al., 2019) and possess superior proliferative and immunomodulatory capabilities compared with other mesenchymal stem cell sources (Ceccarelli et al., 2020) and could be an attractive modality to generate functional implantable tissue for the treatment of cartilage defects (Rahbar Saadat et al., 2020). However, the efficiency of chondrogenic conversion has proved not to equalize other ADSC sources (such as bone marrow or cord blood) (Wei et al., 2007). Derived from perinatal tissues (namely umbilical cord), Wharton Jelly is as a rich source of allogeneic MSCs with superior proliferative and immunomodulatory capabilities (Esposito et al., 2013). Chondrogenic conversion of Wharton jelly-derived mesenchymal stem cells (WJMSC) was shown to be superior to bone marrow mesenchymal stem cells (BM ADSC). Mechanic stimulation of ADSC was shown to increases differentiation to musculoskeletal lineages (bone, cartilage, skeletal muscle) and efficiently increased ADSC as well as WJMSC chondrogenic potential *in vitro* and *in vivo* (Zha et al., 2021). Previous studies have shown that different types of mechanical loading (such as compression, perfusion, vibration, stretching) are effective in increasing ADSC differentiation (Yong et al., 2020). Delivering the appropriate micro-mechanical stimulation that closely mimic the intensity, duration and orientation of mechanical cues in the developmental niche, at cellular level, has proved to be a challenge for musculoskeletal tissue engineering. Mechanical stem cell preconditioning using biophysical stimulation by applying various types of mechanical stress within dynamic bioreactors (compression, shear stress, and hydrostatic pressure) requires ancillary equipment and often a direct contact with the cells or cell media. This introduces supplementary steps in the process of cell manufacturing when intending clinical application. Moreover, at a cellular level, the distribution of applied forces might be uneven with consequences on quality and reproducibility of chondrogenic conversion (Fahy et al., 2018). Alongside with the perspective of introducing a method that waves the need of supplementary equipment, magneto-mechanical stimulation delivers biomechanical cues at a cellular level, more closely reproducing the natural biomechanics. In plus, the enhancement of the chondrogenic differentiation obtained within a magnetic field has also been demonstrated to having a synergistic effect with biochemical factors delivered by differentiation media resulting in an enhanced chondrogenic differentiation (Amin et al., 2014). The use of MNPs of various structures and coatings has been previously found appealing for regenerative medicine purposes when sought as drug and small molecule delivery vehicles as they have the potential to support regenerative processes *in vitro* and *in vivo* as well as for cell tracking and targeting purposes. Nanoparticle-based manipulation of cell and stem cell fate are recognized as the breakout technology capable to consistently contribute to advancement of biological joint resurfacing toward clinical application (Eftekhari et al., 2020). MNP are particularly appealing for manipulating cell fate due to their excellent biocompatibility, versatility and magnetic proprieties (Wimpenny et al., 2012). Of particular interest when using MNP is the ability to use magnetic actuation as a modality to deliver micromechanical stimulation to differentiating cells (Zhang et al., 2020). Mechanical stimulation consistently increase chondrogenic conversion in ADSC. However, the modality to deliver mechanical preconditioning are difficult to translate for potential clinical applications (O’Conor et al., 2013). Iron oxide MNP internalized by ADSC preserve their proliferative and differentiation capability while inducing cell magnetization. This is opening fascinating possibilities for remote cell manipulation under magnetic field (MF) aiming for MNP mediated cell actuation. Such particularity can be used as a modality to deliver remote micromechanical stimulation to stem cells differentiating to musculoskeletal lineages—osteoblasts and chondrocytes (Lima et al., 2015).

Due to their magnetic responsiveness, cells loaded with MNP can be traced within living systems using clinically available MRI imagistic equipment or incoming magnetic particle imaging technologies. The magneto-mechanical effect adds to this already versatile stem cell-MNP functionality the ability to potentially control cell fate by improving differentiation to mechanosensitive lineages, especially those required for musculoskeletal regeneration. In our previous studies we have tested the effect of proprietary uncoated MNP in influencing osteogenesis and adipogenesis and found that magneto-mechanical stimulation increase osteogenesis and decrease adipogenesis in primary ADSC. Here we aimed to test comparatively the effect of magneto-mechanical stimulation in human primary ADSC and WJMSC chondrogenesis. The purpose was to test if MNP we are manufacturing can be used as a modality to deliver magneto-mechanical stimulation to stem cells undergoing chondrogenic differentiation *in vitro* and to identify the most appropriate cell source aiming future cartilage engineering strategies.

Remote magnetic actuation of ADSC and WJMSC, with or without MNP, in alternating magnetic field (MF) was investigated as a modality to deliver mechanical stimulation. Its effect on chondrogenic conversion was assessed quantitatively in order to detect the most appropriate cell source for cartilage engineering purposes.

## Methods

### MNP Manufacturing and Characterization

**MNP.** Reagents used for obtaining Fe_3_O_4_ magnetite MNP were ferrous chloride tetra hydrate (FeCl_2_·4H_2_O, 98%), ferric chloride hexahydrate (FeCl_3_·6H_2_O, 98%), sodium hydroxide (NaOH, 98%) and hydrochloric acid (HCl, 37%) (Sigma-Aldrich). The reagents were used as purchased, without any further purification.

The MNP synthesis followed an in-house modified hydrothermal approach as previously reported for this study. 2.2 g FeCl_2_·4H_2_O were dissolved in 4 ml deionized water (DW) and mixed with 6 g FeCl_3_·6H_2_O. After filtering through a 220 nm filter, the iron salt solution was mixed with 1 ml HCl and added into 200 ml boiling DW. After mixing with solid NaOH (15 g) under magnetic stirring (1,300 rpm), the solution turned immediately black. The solution was further mixed for 30 min (1,000 rpm), whereas the heating was turned off. MNP were magnetically separated and submitted to several washing steps until the pH of the suspension reached 6.5.

**Magnetic characterization.** The magnetization data were acquired using vibrating sample magnetometer (VSM) (LakeShore 7410) in magnetic fields ranging between −20 and 20 kOe for both bare MNP and WJMSC/ADSC MNP. The morphology and size of MNP was determined through transmission electron microscopy (TEM) (Libra 200 UHR-TEM, Carl Zeiss, Germany). Assessment of the Zeta potential was performed with Malvern Zetasizer NS (Malvern Instruments).

### Human Primary Adipose Derived Stem Cells

Human primary ADSC were obtained from healthy donors undergoing elective cosmetic liposuction procedures after institutional ethical approval and informed patient consent. Lipoaspirate was processed within maximum 24 h after surgical procedure (average 3 h) as previously described (Labusca et al., 2018). Briefly, lipoaspirate was washed with PBS, digested with collagenase type I (0.01 mg/ml) for 2 h at 37.5°C, and centrifuged at 300 g for 5 min at room temperature. The supernatant was removed and the medium further centrifuged at 300 g for 5 min. Pelleted cells were re-suspended in complete culture media (CCM) (DMEM, 10% FBS, 2% Antibiotic/antimycotic). Cells were automated counted (TC20™ Automated Cell Counter, Bio-Rad Hungary Ltd.) and plated at 1 × 10^6^ cells/cm^2^ in tissue culture flasks (CellBIND surface, Corning). Passage three to four cells were used for experiments. For morphology evaluation, the cells were observed under a fluorescent inverted microscope (EVOS Fl Life Technologies).

### Human Primary Wharton Jelly Mesenchymal Stem Cells

Umbilical cord from healthy parturient at term birth was collected after signed informed consent and transported to the laboratory in the first 4 h after delivery. Wharton Jelly was carefully dissected from blood vessels, washed with antibiotic solution, minced with a scalpel in pieces of 1–1.5 mm width, placed in six well plates in complete culture media and kept in the incubator. After 5–7 days, the explanted cells were removed using Trypsin/EDTA and re-plated in appropriate tissue culture flasks. Cells passage two to four were used in the experiments for this study.

### ADSC and WJMSC Loading With MNP, Viability and Proliferation

For cytotoxicity assays, ADSC and WJMSC were plated in 96 well plates, at 2 × 10^4^ cells/well and incubated for 48 h. MNP in concentration of 40 μg/ml were added to complete culture media and further incubated for 24 h. MNP suspension was delivered once for all cell types and all experiments without any further addition. Cell viability tests were performed using MTT (5-dimethylthiazol-2-yl-2, 5-diphenyltetrazolium bromide-Vibrant ®TermoFisher Scientific) assay according to supplier’s instructions. Absorbance was read at 570 nm. Cell viability (CV) as expressed by MTT optical density (OD) was calculated using the formula CV = 100 × (ODs-ODb)/(ODc-ODb), where ODs = OD of particle treated cells; ODb = OD of blank (media only); ODc = OD of untreated cells. Cell viability and proliferation was investigated at three time points (2, 5 and 10 days) using MTT as well as cell counting after fixation with 80% ethanol and nuclear staining with 4′,6-diamidino-2-phenylindole (DAPI) Termofisher Scientific) 0.1 μg/ml for 5 min, washed twice. Cell count/well 10) was performed using a fluorescent microscope and a grid using the formula X = 2*A/(L*L)*N where A is the surface of the well, L is the size of one square in the grid, N the number of cells per field of view (FOV). Averaged count for at least five FOV were counted/experiment. Cell proliferation expressed as population doubling (PD) was calculated using the formula PDn= [LOG (Nn)-LOG (N0)]/LOG (2) where, N is the number of cells counted at the end of each interval, n represent the time point (day 2, 7 and 14 of cell culture) and N0 number of viable plated cells.

### Quantitative Assessment of MNP Content - Ferrozine Assay

ADSC and WJMSC were plated in 24 well plates at 1 × 10^5^ cells/well. MNP were added after 24 h, and the experiments were performed in four replicates. At 1, 7, 14 and 28 days of cell-particle interaction, respectively, the cells were double washed with PBS to remove any extracellular MNP, fixed with 70% ethanol for 15 min and double washed with PBS. 500 μL of 50 nM NaOH were added to three wells per experiment for 2 h on a shaking plate. Aliquots of cell lysates were then transferred to 1.5 ml Eppendorf tubes and mixed with 500 μL of 10 mM HCl, and 500 μL of iron-releasing reagent (a freshly mixed solution of equal volumes of 1.4 M HCl and 4.5% (w/v) KMnO4 (Merck, Germany) in distilled H2O. These mixtures were incubated for 2 h at 60°C within a fume hood. 150 μL of iron-detection reagent (6.5 mM ferrozine (Sigma-Aldrich, St Louis, United States), 6.5 mM neocuproine (Sigma-Aldrich), 2.5 M ammonium acetate and 1 M ascorbic acid (Sigma-Aldrich) dissolved in water) were added to each tube. After 30 min, 500 μL of the solution obtained in each tube was transferred into a well of a 24-well plate, and absorbance was measured at 570 nm with a spectrophotometer. A calibration curve was set up using FeCl_3_ standards (0–300 µM) in 10 mM HCl to allow calculation of iron content per sample.

### Cell Senescence

ADSC, WJMSC, ADSC-MNP and WJMSC were seeded in 24 well plates at 4 × 10^5^cells/well. Beta galactosidase enzyme activity was assessed using β-galactosidase (B-Gal) Detection Kit (Fluorometric) (Abcam) accordingly to manufacturer instructions. Briefly, cells at 14 and 28 days of culture were lysed using lysis buffer, afterwards Fluorescein di-β-D-Galactopyranoside (FDG) stock solution was added to each well and incubated for 1 hour at 37°C, treated with stop buffer solution to increased FDG fluorescence intensity and read using a microplate reader (490–550 nm excitation/emission). Cell number was calculated at each time point as described for ferrozine assay.

### Assessment of ADSC and WJMSC Cytoskeleton Fibers in Magnetic Field

2 × 10^5^ cells (passage 3–5) were plated in 35 mm Petri dishes in DMEM with 10% FBS. After 24 h, cells were treated with 40 μg/ml MNP and further cultivated for 24 h. For generation of magnetic field, we used an in house-built system based on four crossed coils programmed to generate a rotating magnetic field (MF) as previously described (Chiriac et al., 2018). ADSC and WJMSC with or without MNP were exposed to MF for 20 min; cells were fixed with 2% paraformaldehide for 15 min, stained with phalloidin (Texas Red™-X PhalloidinTermo Fischer Scientific) and counterstained with DAPI Images were taken using a fluorescent inverted microscope (EVOS Life Technologies).

### Chondrogenesis

For *chondrogenesis assays*, 9 × 10^5^ ADSC, WJMSC, ADSC-MNP and WJMSC-MNP were pelleted in incomplete chondrogenic media (ICM) composed of - DMEM (high glucose -HG), Dexamethasone 1mM, Ascorbic acid 2-P: 5 mg/ml L-Proline: 4 mg/ml ITS + supplement Sodium pyruvate). After pelleting, ICM was changed with complete chondrogenic media (ICM plus TGFβ-3 10 ng/ml). Chondrogenic pellets were kept in 15 ml polypropylene tubes in incubator and fed twice/week for 21 days.

*Assessment of chondrogenesis*. After 21 days, pellets were fixed in 10% formaline solution for 30 min, washed twice with PBS and embedded in paraffin for histological evaluation. Sections were stained with Safranine O 0.1 ml/L. For quantitative chondrogenesis evaluation, pellets were digested using papain and total amount of GAG glycozaminoglycans content was assessed using chondroitin-6-sulphate DMMB (1, 9 Dimethylmethylene blue) method. DMMB absorbance was read using a microplate reader. The results were normalized against pellet total DNA content obtained QuantiTPico Green dsDNA kit (Invitrogen).

### ADSC and WJMSC Differentiation in Magnetic Field

Test tubes with chondrogenic pellets were positioned in the center of the coil and subjected to alternating MF for 10 min every 2 h in the first 7 days after chondrogenic induction, Temperature of the samples was measured using an optical fiber thermometer. Non exposed controls were kept in the incubator.

**Statistical analysis**. Cells from four donors were used in this study. At least 5 technical replicates were used in each experiment per donor. One way analysis of variance (ANOVA) was performed using OriginLab PRO version 9.0 followed by Bonferroni post hoc analysis; between-group and within group differences that were considered significant at *p* ≤ 0.05 (*n* = 5).

## Results

### Magnetic Nanoparticle Characterization

**FIGURE 1:**
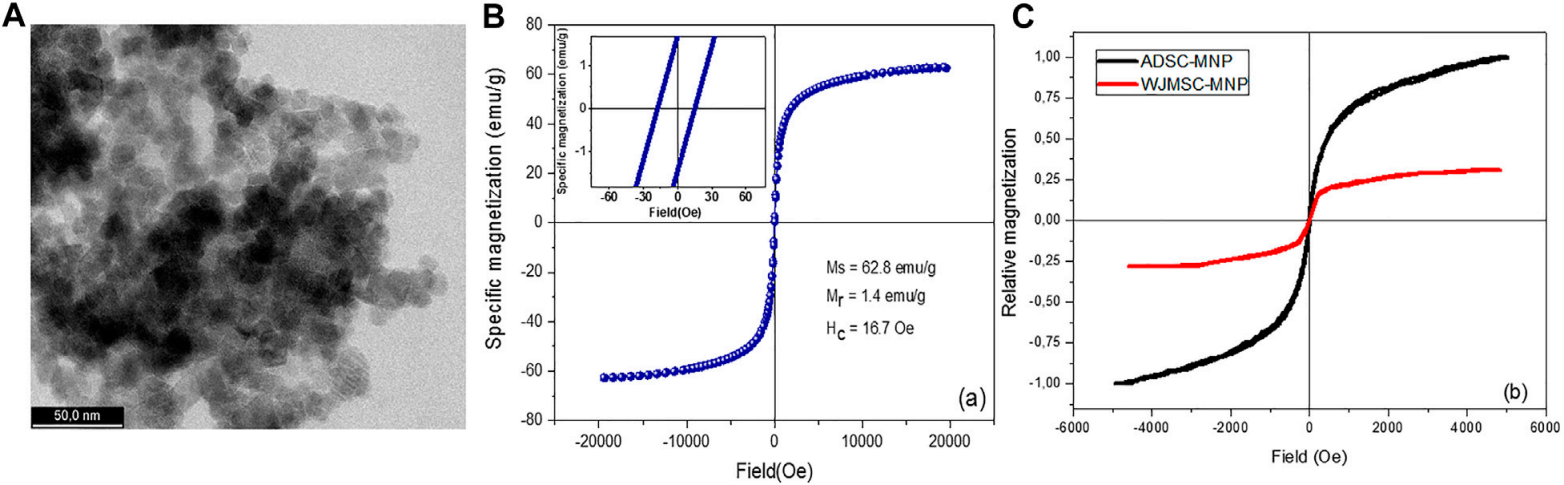
Ultra-high resolution transmission electron microscopy (UHR-TEM) demonstrating the size of Fe_3_O_4_ nanoparticles **(A)**; magnetization loop of bare magnetite nanoparticles [**(B)**-inset: details from the low-field region]; magnetization loops of WJMSC and ADSC loaded with magnetite nanoparticles **(C)**.

High-resolution TEM (HRTEM) imaging have shown MNP with dimensions ranging between 10 and 15 nm with a fine size distribution. MNP are shown to having different shapes such as cubic, octahedral, or spherical (Figure 1A).

Zeta potential of the MNP was found to be −32.72 (±1.27), pointing out an excellent stability in aqueous solutions. The strong electrostatic repulsion most probably overcome the remanent magnetization of the MNP. An excellent stability was empirically observed for the MNP aqueous suspensions which remained stable for about 2 months. However, following a short ultrasonication, MNP disperse very well, recovering their initial stability. The hysteresis loop of bare magnetite, obtained for magnetic fields up to 2 T, followed a profile specific to ferromagnetic materials. The relatively low remanent magnetization, Mr, and coercive field, Hc, allow the MNP to diminish the agglomeration induced by magnetic attraction (Figure 1B).

### Short and Medium Term ADSC-MNP and WJMSC Viability

**FIGURE 2:**
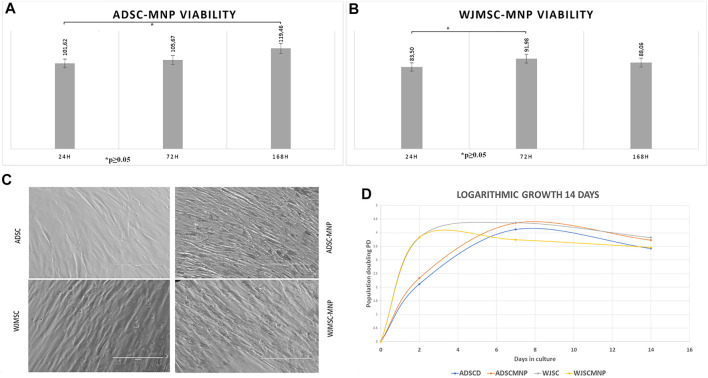
Long term viability in culture MTT assay for cells exposed to 40 μm/ml Fe_3_O_4_ bare MNP added in the cell culture media; ADSC demonstrate significant increase in MTT activity at 7 days (168 h) compared to 24 h: at all time points viability is more than 100% of non-loaded cells demonstrating good overall viability and lack of MNP cytotoxicity **(A)** WJMSC viability has a minimum at 24 h cell particle interaction increase at 72 h compared to 24 h, slightly decreases at 7 days demonstrating acceptable viability and MNP toxicity **(B)**; morphological evaluation of ADSC-MNP compared to ADSC and WJMSC compared to WJMSC-MNP after 48 h cell particle interaction demonstrating maintenance of cell shape, apparent membrane integrity and even distribution of MNP inside cell body without penetrating nuclei with visible higher amount of incorporated MNP for ADSC-MNP than WJMSC-MNP **(C)**; logarithmic growth curve of MNP loaded and non -loaded ADSC and WJMSC Cell proliferate, with lower, non-significant PD values for WJMSC-MNP and higher, non-significant PD for ADSC-MNP compared to ADSC and WJMSC up to 7 days and plateau afterwards in order to displayed a slight descendent trend at day 14 compared to day 7 most probably due to reaching confluence in the culture dish **(D)**. PD = population doublings.

ADSC and WJMSC were adherent to tissue culture plastic, displayed volume positivity for CD 105, 76 and 90 and negative for CD 34 and 45 (Supplementary Charts S1, S2). All cells underwent three lineage differentiation (chondrogenesis as below, adipogenesis and osteogenesis–Figures 1, 2 Supplementary Material) (Dominici et al., 2006).

Viability of ADSC and WJMSC loaded with MNP, starting 24 h after cell-particle exposure, was tested against non-loaded cells using the MTT assay. This specific assay is based on the quantitative colorimetric assessment of tetrazolium reduction to formazan salt by the mitochondrial dehydrogenase activity in viable and non-quiescent cells, an indirect estimate of mitochondrial metabolic activity and cell viability. Excellent viability was recorded for ADSC-MNP after short and medium term ADSC-MNP interaction (24, 72 h and 7 days–164 h) (Figure 2A). A significant time dependent increase in relative dehydrogenase activity between 24 h and 7 days can be attributed to increased ADSC-MNP proliferation compared to non-loaded cells. WJMSC-MNP displayed satisfactory viability after exposure to MNP. The maximum WJSC-MNP viability (91.9% from non-loaded cells) was recorded at 72 h. Unlikely ADSC-MNP, whose relative viability continued to increase in time, WJMSC-MNP viability decreased non-significantly at 7 days and 14 days (Figure 2). No visible morphological changes regarding cell shape or membrane integrity could be observed for both cell types after exposure to MNP (Figure 2C). ADSC-MNP displayed good to excellent viability over extended time in culture (up to 7 days) and continued to proliferate, displaying more than 100% viability at all time points. WJMSC displayed reduced viability in same culture conditions with a minimum at 24 h (83% viability of non-loaded WJMSC), suggesting MNP presence influence their viability, increasing at 72 h.

### ADSC-MNP Proliferation and Senescence

Since MTT results suggested an increase in cell number per well in ADSC-MNP, we further tested the comparative proliferative activity of ADSC-MNP and WJMSC. Logarithmic cell growth was calculated based on number of cells counted after fixation with ethanol 80% and nuclear staining with DAPI. Cells were counted using an inverted fluorescent microscope, the number of cells from at least 5 FOV were averaged and the results were used to determine the number of cells/well (see the methods). ADSC-MNP displayed slightly increased population doubling (PD) compared to non-loaded cells up to 14 days in culture while WJSC-MNP showed decreased proliferation at 7 days compared to non-loaded cells. All cell types continued to proliferate, with lower, non-significant PD values for WJMSC-MNP and higher, non-significant for ADSC-MNP compared to their respective non-loaded counterparts up to 7 days and starting to plateau afterwards most probably reaching confluence in the culture dish. All growth curves displayed a slight descendent trend at day 14 compared to day 7. (Figure 2D).

**FIGURE 3:**
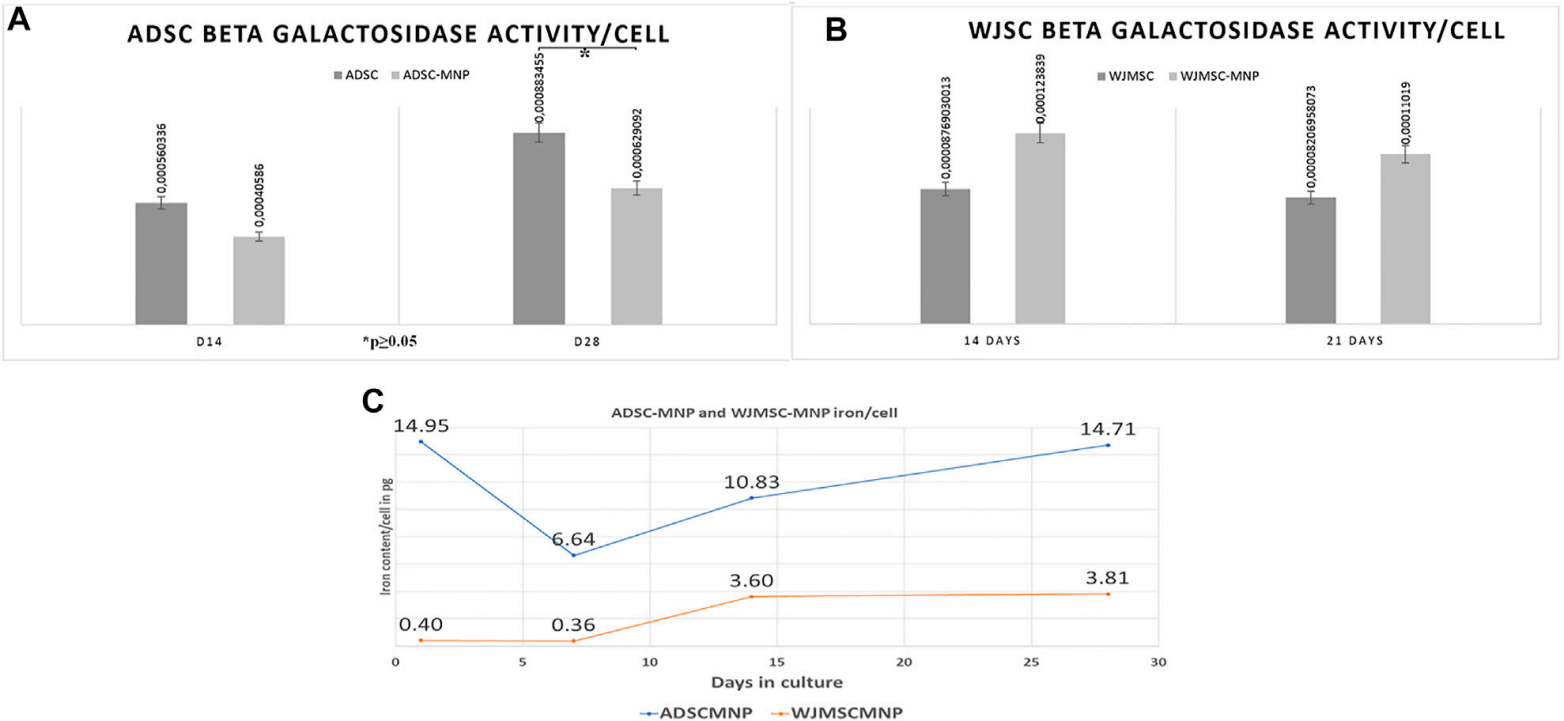
Beta galactosidase activity (B-Gal) in ADSC **(A)** and WJMSC **(B)** demonstrating significant less enzymatic activity in ADSC-MNP at 28 days in culture compared to ADSC at same time point **(C)**; iron content/cell (in picograms) at 1, 7, 14 and 28 days cell-particle interaction; after 24 h cell particle interaction ADSC aquire enough iron content to become remote controllable within MF (14, 0 pg/cell).

For detection of cell senescence in culture we used quantitative assessment of β-galactosidase (B-Gal) activity calculated per cell. Results revealed lower enzyme expression in ADSC-MNP at 14 days as well as significant lower B-Gal activity in ADSC-MNP after 28 days in culture compared to non-loaded ADSC (Figure 3A). WJMSC-MNP however displayed increased B-Gal activity at both time points in culture compared to WJMSC at similar time points (Figure 3B).

### Iron Content per Cell

The quantity of iron load represented by the up-taken MNP was calculated by subtracting iron content of non-loaded cells from ADSC-MNP and WJSC-MNP respectively. Iron content was determined spectrophotometrically using the ferrozine assay. After 24 h of cell particle interaction, ADSC acquire an average amount of 14.9 pg iron/cell (3.67 × 10^6^ MNP/cell), decreasing to 6.64 (1.63 × 10^6^ MNP/cell) at 7 days in culture. Interestingly, at 14 days the amount of iron/cell was found to be increased to 10.8 pg (1.63 × 10^6^ MNP) in order to reach 14.75 pg/cell (3.62 × 10^6^ MNP) at 28 days, almost similar with content at 24 h. WJMSC-MNP iron load was significantly lower at 24 h (0.4 pg/cell; 9.8 × 10^4^ MNP/cell) and 7 days (0.36 pg/cell; 8.8 × 10^4^ MNP/cell) compared to ADSC-MNP at same time point and reached 3.6 pg/cell after at 14 days and 3.81 at 28 days in culture, respectively (Figure 3C).

### ADSC-MNP and WJSC-MNP Magnetization

For ADSC and WJMSC loaded with MNP, a marked difference is underlined by the hysteresis loops (Figure 1C). The amount of MNP uploaded by WJMSC is very low compared to ADSC, as already confirmed by quantitative evaluation of iron content (see above). ADSC-MNP upload an increased amount of MNP in similar condition (14.95 pg iron/cell) within 24 h of cell particle interaction and culture media preconditioning of MNPcompared to WJMSC (0.4 pg iron/cell). ADSC-MNP become responsive to applied magnetic field while WJMSC display low MF responsiveness and magnetization in similar culture conditions and duration of cell particle interaction.

### Qualitative Assessment of ADSC Cytoskeleton Fibers in MF

**FIGURE 4:**
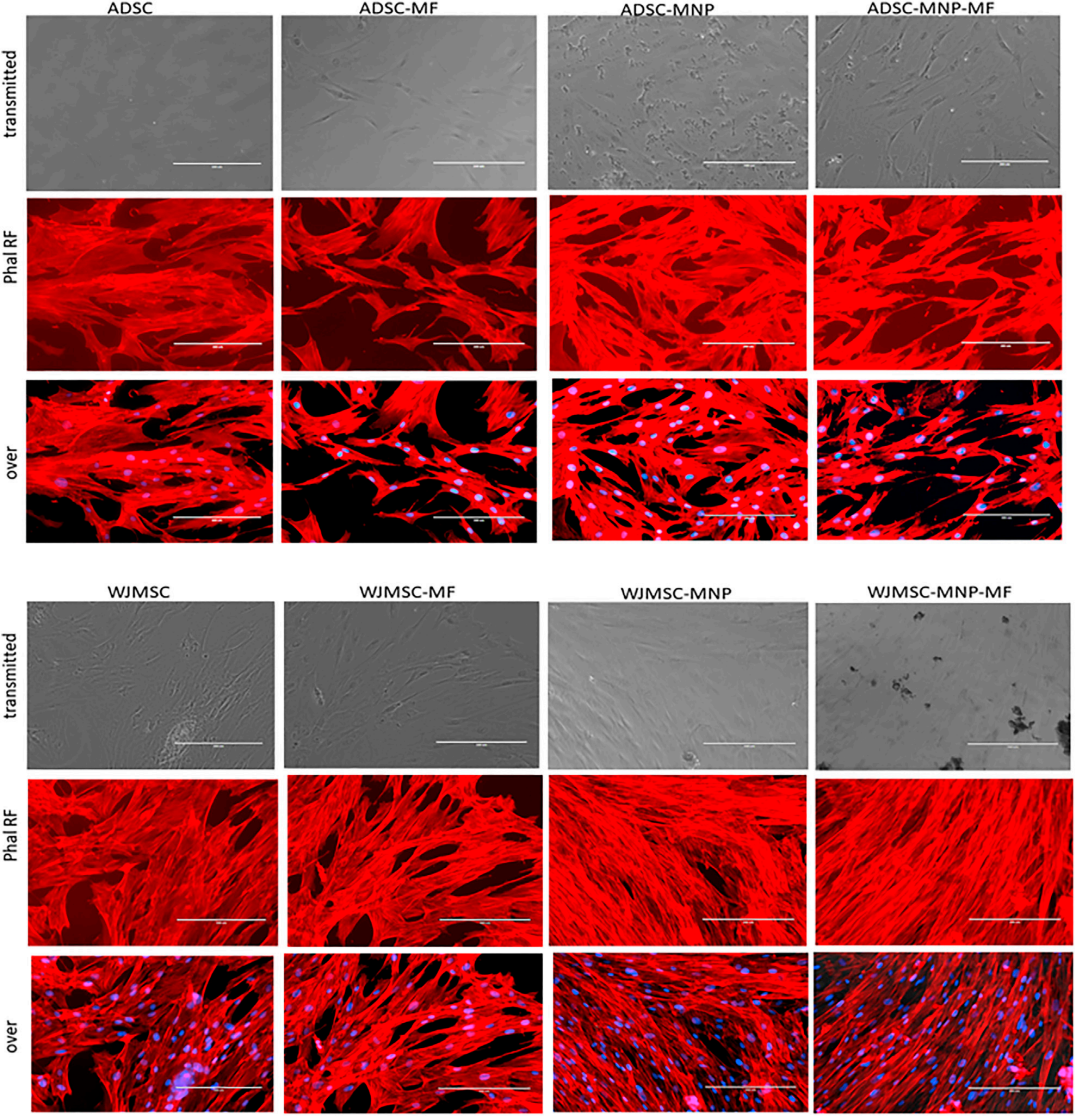
Cytoskeletal fibbers in loaded and non-loaded ADSC and WJMSC demonstrating qualitatively actin fibbers shrinkage in cells exposed to MF, especially for MNP loaded cells. Texas Red™-X Phalloidin staining, nuclei stained with DAPI; EVOS inverted fluorescent microscope 20 × magnification.

Both loaded and non-loaded ADSC and WJMSC exposed to 6 mT alternating MF for 20 min displayed distortion of actin fibers. Cell body became edgy and uneven compared to non-exposed cells. Furthermore, in ADSC-MNP exposed to MF, the cytoskeletal fibers became shrunk with multiple edges, while for the WJMSC-MNP this effect was not visible. No obvious distortion of cell nuclei could be observed. (Figure 4).

### Chondrogenic Conversion

**FIGURE 5:**
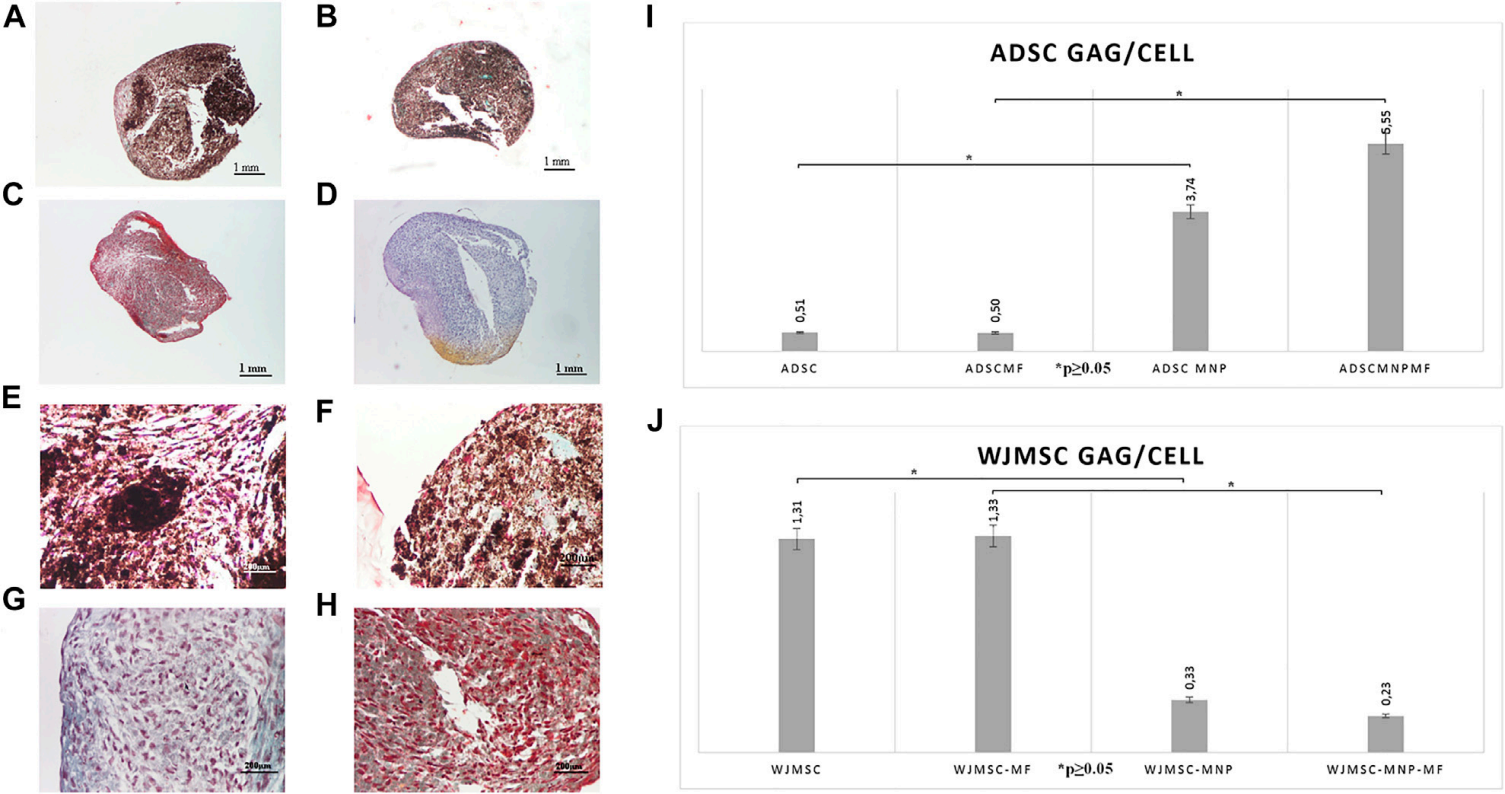
Chondrogensis assay: qualitative: representative histological images of Safranin O stained pellets formed by ADSC-MNP-MF **(A)**; ADSC-MNP **(B)**; ADSC-MF **(C)**; ADSC **(D)** A-D Nikon Eclipse E600 inverted microscope ×4 magnification; ADSC-MNP-MF **(E)**; ADSC-MNP **(F)**; ADSC-MF **(G)**; ADSC **(H) (E–G)** Nikon Eclipse E600 inverted microscope ×20 magnification; quantitative evaluation of glycosaminoglycan (GAG) deposition/cell/pellet in ADSC demonstrating significant increase in GAG deposition in MNP loaded cells as well as in MNP loaded cells submitted to magnetomechanical stimulation within MF compared to non loaded cells **(I)**; WJMSC quantitative evaluation of GAG deposition/cell/pellet demonstrating significant increased GAG deposition in non loaded WJMSC; to note, the maximum amount of GAG deposition is displayed by ADSC-MNP-MF in similar experimental conditions **(J)**.

Pelleted ADSC, ADSC-MNP, WJMSC and WJMSC-MNP were treated with chondrogenic differentiation media for 21 days. MF exposure was performed by exposing test tubes to MF for 10 min every 2 h during the first 7 days after chondrogenic induction. Pellets formed from MNP-loaded and MF-exposed cells are more compact, dense and rounded (Figures 5A,B) compared to non-loaded (Figures 5C,D). Iron presence inferred brownish overall aspect of the pellet, still the characteristic pink color represented by acidic proteoglycan staining could be detected with higher magnification (Figures 5E,F). Chondrogenic differentiation was investigated using quantitative colorimetric GAG assay and the results were normalized to cell number/pellet. Cell number/pellet was obtained by dividing DNA content/pellet expressed in pg to 6, the approximated quantity of cellular DNA (Gillooly et al., 2015). In order to exclude a potential interference in colorimetric evaluation of GAG content, for the case of MNP-loaded cells, calculation of GAG content was performed using a standard curve raised for chondroitin sulfate mixed with MNP. The amount of MNP added to each CS dilution was calculated as the iron content per cell resulted from ferrozine assay multiplied by the number of cells/pellet. ADSC-MNP displayed significant increase in GAG content per cells compared to ADSC. Furthermore, ADSC-MNP exposed to MF displayed significant increased GAG/cell ratio compared to non-loaded cells. (Figure 5I,J). MF exposed WJMSC and non-exposed WJMSC generated significantly higher amount of GAG/cell compared to WJMSC-MNP and to WJMSC-MNP in MF. Remarkably, the maximum amount of GAG/cell generated by ADSC-MNP-MF (5.55 pg) was almost five times higher compared to maximum amount generated by WJMSC-MF (1.33 pg).

## Discussions

The ability to produce bioengineered tissues and organs relies on abundant cell sources that have the capability to convert to the desired lineage under appropriate biochemical and mechanical cues. Mesenchymal stem cells have been intensively sought for tissue engineering of cartilage and a large number of research groups are reporting promising *in vitro* and *in vivo* results. The importance of mechanical stimulation for obtaining functional engineered bio equivalents especially in the case of musculoskeletal tissues has been stressed out (Athanasiou et al., 2015). Various methods for mechanical stimulation and dynamic bioreactors have been designed for this specific purpose. However, the ancillary equipment for delivering mechanical load is in many cases bulky, resource consuming and difficult to scale up for translational purposes. We have previously reported good results using magneto-mechanical stimulation for osteogenic and chondrogenic conversion of human primary ADSC using incorporated proprietary MNP and MF exposure. ADSC and WJMSC are considered efficient cell sources for tissue engineering due to their good proliferative and differentiation capabilities, easiness of access and lack of ethical issues with their procurement. WJMSC of allogeneic origin are reportedly non-immunogenic, can generate clinically relevant number of mesenchymal progenitors and are already the object of clinical trials (Liang et al., 2020). In this study we focused on comparing the two cell sources in respect to their capability to undergo chondrogenic lineage differentiation under magnetically delivered mechanical stimulation.

Proprietary bare magnetite MNP are prepared by co-precipitation of iron salts, method that enables production of relatively homogenous nanoparticles (Perlstein et al., 2010). MNP used in this study have average size of 20 nm and display ferromagnetic proprieties and characteristic magnetization curve under MF exposure. Due to their reduced size, sterilization by autoclave induce particle aggregation (data not shown). Therefore, we used for sterilization UV exposure in the biosafety hood. MNP, in fixed concentration of 40 μg/ml, were first diluted in complete culture media in order to allow formation of a protein corona. Since the protein corona is the major factor that determines MNP biological identity, “presentation” to cell membrane and their internalization (Nierenblerg et al., 2018), one single batch of FBS was used for all experiments in this study for consistency. Simple addition in culture media resulted in different behavior of MNP in relation to the two cell types investigated here. In the first 24 h of cell-particle interaction, MNP were observed to agglomerate around WJMCSs without being internalized. With increasing time in culture, particles were observed in the pericellular area as well as inside cytoplasm. Conversely, MNP could be detected inside ADSC cytoplasm as soon as 24–48 h and further on through all duration of observation (maximum 28 days).

The particularity of WJMSC-MNP interaction could be probably explained by the presence of a dense pericellular mesh (Wang et al., 2018) that interfere with initiation of endocytosis. At the initial contact with the cells, MNP are firstly interacting with pericellular matrix and plasma membrane and uploaded by endocytotic mechanisms that vary with their size, coating, and electric charge. MNP are included within intracellular vesicles and finally degraded in the acidic environment of the endo-lysosomal compartment (Kolosnjaj-Tabi, 2013). Bare and as well Fe_3_O_4_ MNP coated with biocompatible shells are known to be nontoxic, non-genotoxic and not to induce hemolytic processes in contact with blood (Fan et al., 2020). Previous reports have shown good viability in many stem cell types upon internalization of commercially available MNP with different coatings (Wimpenny et al., 2012). Our group has reported good viability in various human stem cells (bone marrow, trabecular bone, adipose derived) with proprietary bare, chitosan and palmitic acid coated Fe_3_O_4_ MNP. A particularity of MNP that distinguish them from other nanoparticles is the existence of a cellular mechanism for iron storage and metabolism. Ferritin-dependent iron homeostasis is an endogenous mechanism ubiquitous and evolutionary preserved in mammalian cells. Ferritin protein cages accommodate MNP degradation products while concomitantly slowing down the degradation process due to the colloidal behavior in acidic medium (Volatron et al., 2017). A dose dependent reduction in WJMSC viability and proliferation after exposure to commercially available MNP as assessed using MTT was reported (Ohki et al., 2020) suggesting that the maximum iron load the preserves phenotype is cell type dependent. The correlation between maximum iron load that preserves viability and specific cytosolic ferritin activity in various cell type would warrant further investigation. We found that viability of WJMSCs exposed to MNPs after 24 h was only 83,5% compared to non-loaded control as assessed by cell mitochondrial activity. While this is not obligatory a proof that MNPs are cytotoxic to WJMSCs, it is revealing limited mitochondrial activity compared to non-loaded. This could be possible explained by the fact than iron handling enzymatic equipment (especially mitochondrial ferritin) as well as iron overload threshold is cell specific. Mammalian cells response to similar amounts of iron traffic and load is known to not only dependent on cell type but as well on cell status and activity (Ward and Cloonan, 2018). Further investigatings are needed to explore this particularity.

As resulting from population doublings, both cell types, loaded and non-loaded, continue to proliferate up to 14 days when they reach a plateau, most probably due to culture conditions (reaching over confluence). ADSC-MNP display slightly higher PD compared to non-loaded while ADSC-MNP slight decrease proliferation upon MNP exposure. Iron is known to play a crucial role in energy metabolism, being constituent of mitochondrial enzymes of the electron transport chain, involved in ATP production, oxygen consumption, cell cycle progression and proliferation. Excess of iron, however, generate Fenton reaction producing reactive oxygen species (ROS), especially hydroxyl radicals which can have destructive effects on cellular lipids and DNA (Wessling-Resnick, 2017). The delicate balance between promoting mitochondrial activity and cell proliferation and inducing ROS formation that arrest cell cycle is dependent on complex factors of which free iron ions formation, and availability of intracellular ferritin. Expression of iron transport and metabolism proteins in ADSC, as well as activation of ROS-stimulated pathways are likely to be dependent on tissue of origin, donor age, presence or absence of associated disease (diabetes mellitus, obesity) (Mehta, 2021). Here we found that even if the amount of iron accumulated per cell in the first days in WJMSC is significant lower compared to ADSC-MNP, their growth rate is non-significantly decreased. WJMSC are isolated from WJ, a tissue originating from extraembryonic mesoderm that functions as the very first hematopoietic niche in the developing embryo. WJMSC were shown to support hematopoiesis *in vitro* (Lo Iacono et al., 2018) and to possess a particular transcriptome compared to other ADSC (Jeong et al., 2021), possibly being less equipped for iron internalization. Adenoviral-mediated ferritin overexpression for WJMSC performed as a method to induce their traceability using magnetic resonance imaging (MRI) equipment was found to significantly decrease cell proliferation. (Dai et al., 2017). Further investigation of WJMSC particularities regarding iron transport and metabolism would be necessary.

Bare MNP could be uploaded by ADSC, but not by WJMSC within first 24 h. The average amount of 14.9 pg iron load per cell unit induced efficient cell magnetization in ADSC-MNP suspension as demonstrated by VSM. In WJMSC, the amount of up-loaded iron was significantly lower; however, cells still displayed a magnetic behavior, demonstrating they could respond to an applied MF. This finding was further supported by the configuration of cytoskeletal fibers in loaded and non-loaded cells exposed to MF. After brief exposure (10 min) to a relatively low intensity MF (6 mT), F actin fibers showed the tendency to disorganize and to distort cell contour. Static field exposure (1 mT) of dental pulp stem cells (DPSCs) was shown to rearrange cytoskeleton and to increase their migration proliferation and differentiation by recruiting YAP/TAZ to the nucleus, inhibiting its phosphorylation and upregulating two YAP/TAZ-regulated genes, CTGF and ANKRD1. MF induced YAP/TAZ nuclear localization was inhibited by cytoskeleton inhibitor, cytochalasin D (Zheng et al., 2018). In our study ADSC-MNP and, at a lower extent, WJMSC-MNP displayed increased cytoskeletal fiber rearrangement under MF (Figure 4). MNP are known to be trafficked inside cell cytoplasm along cytoskeletal fibbers. It is possible their proximity to actin fibbers during MF exposure to generate micro movements that act as internal mechanical stimulation, influencing YAP/TAZ nuclear translocation and possible other mechano-responsive pathways.

An interesting observation is the particularity of iron load dynamic on the duration of 28 days in ADSC-MNP. After 24 h cell-MNP interaction, cells were found to accumulate the maximum amount of iron/cell (14.9 pg/cell), quantity that was found to decrease to almost one third (6.64 pg/cell) at 7 days, then increased again to reach approximatively the initial amount at 28 days (14.71 pg/cell). The initial decrease in iron content at 7 days can be partly explained by proliferation and consequent distribution of MNP in the dividing cells. Other possible explanations such as particle biodegradation and re-synthesis could be involved. Using magnetism as a modality to demonstrate the MNP presence inside cells, a possible mechanism of biodegradation and “re-biosynthesis” was demonstrated to occur in human ADSC. Cells were found to be “re-magnetized” after degradation of nanoparticles that took place at 7–10 days after particle internalization (Van de Walle et al., 2019). This finding was interpreted as a direct demonstration of a possible neo synthesis of magnetic nanoparticles inside endosomes, a process H-ferritin dependent that could explain, at least in part, the gradual increase in iron content/cell at 14 and 28 days for ADSC-MNP. WJMSC increased iron/cell gradually up to 28 days probably as a result of slow internalization of the MNP due to rich fibronectin pericellular matrix and a possible low expression of transferrin or other iron transporters, fact that needs to be further investigated.

We used assessment of B-Gal activity as a modality to detect culture induced senescence. Not surprisingly, B-Gal expression was found to increase at 28 days in all cell types, loaded and non-loaded, compared to 14 days. However, ADSC-MNP showed less B-Gal activity compared to non-loaded at both time points whereas WJMSC-MNP were found to express increased enzyme activity compared to non-loaded. The presumptive lower activity of cytosolic ferritin and increased ROS formation in WJMSC could be involved. Culture-induced senescence is a well-known phenomenon that impedes *in vitro* cell expansion and negatively influences MSC therapeutic potential (Liu et al., 2020). No significant ROS increase and cell ageing could be observed in human myoblasts loaded with different amounts of superparamagnetic iron oxide nanoparticles (SPIOS) that retained their phenotype *in vitro* and *in vivo* (Wierzbinski et al., 2018). Here we found that non coated MNP internalization decreased B-Gal activity during extended culture in ADSC but not in WJMSC. Further studies focusing on ROS expression as well as on investigating senescence associated phenotype are needed to determine if MNP loading could function as a modality of preventing culture induced senescence in ADSC populations.

Based on quantification of GAG deposition per cell, we found that condrogenic conversion of MF-exposed and non-exposed ADSC-MNP was significantly increased compared to non-loaded. Furthermore, MF exposure increased GAG deposition in ADSC-MNP compared to non-exposed ones, albeit non-significantly. Iron is an essential factor for cartilage formation and its internalization is required for ECM synthesis. Reduced iron is a catalyst of proline and lysine hydroxylation reactions that are essential to the formation of mature collagen molecules (Pacifici and Iozzo, 1988). MNP can provide slow release of reduced iron ions supporting chondrogenic ECM deposition. Stem cells were found to handle iron transport differently depending on differentiation pathways even in the absence of MNP. In MSC chondrogenesis, transferrin is down regulated while ferroportin is significantly higher expressed compared to osteo and adipogenesis. Moreover, ADSC loaded with MNP, overexpressed L-Ferritin (involved in iron storage as nonmagnetic ferrihydrite in the ferritin cage) during chondrogenesis. Conversely, H-Ferritin, possible involved in *de novo* MNP biosynthesis, was found to be higher expressed during osteogenesis and adipogenesis (Van de Walle et al., 2019). Iron availability and supplementation has been recently confirmed as a modality to increase chondrogenesis *in vitro* and *in vivo* in Guineea pigs. Ascorbic acid and iron supplementation in the form of commercially available ferumoxytol was found to significantly increase chondrogenesis in bone marrow ADSC *in vitro* as well as *in vivo* in Guinea minipig model of cartilage defect (Theruvath et al., 2021). Ferrumoxytol, is used as iron supplement for intravenous administration and occasionally off label as a MRI contrast agent for vascular and nodal metastasis. It consists of carbohydrate-coated ultra-small SPIOs (Vasanawala et al., 2016). Their hydrodynamic size (average 25 nm) and iron oxide composition are similar with particles used in our study. Here we used non-coated MNP, fact that could increase intracellular iron availability and decrease the time of MNP processing by the cells. The fact that WJMSC iron handling mechanisms cannot provide similar amounts of intracellular iron compared to ADSC (as resulting from the quantitative assessment of intracellular iron content) can possible interfere with chondrogenic conversion. Profiling comparatively the cellular iron enzymatic equipment in loaded and non-loaded ADSC and WJMSC during chondrogenic conversion could reveal very useful insights potentially offering molecular targets to enabling future cartilage engineering procedures. ADSC chondrogenesis was previously found inferior to other stem cell sources (Fellows et al., 2016). In this study, significant higher amounts of GAG/cell obtained from non-loaded WJMSC compared to non-loaded ADSC, confirming the fact that non-modified ADSC are not the best source when considering cartilage engineering. However, MNP presence clearly increased GAG deposition in ADSC, especially after MF exposure. Actin cytoskeleton was shown to modulate COL2/aggrecan fragment generation; the factors that control actin dynamic decisively control the chondrocyte phenotype (Lauer et al., 2021). MNP presence, in close relation to actin fibbers (Orynbayeva et al., 2015), can induce the necessary actin dynamics to initiate and promote chondrogenesis. In this study, we showed that MF exposure influences the cytoskeletal fiber organization. GAG deposition in magnetically stimulated ADSC-MNP was almost 5 times higher compared to non-loaded WJMSC, pointing to a possible modality for manipulating ADSC for improved chondrogenic conversion *in vitro* and, possibly, future methods for improving cartilage-repair pulsed magnetic field exposure (PEMF) of BM-ADSC in combination with magnetic hydrogels which were shown to increase repair in rabbit model of cartilage defects (Huang et al., 2019). Further studies are needed to more precisely clarify the molecular mechanism governing the correlation between MNP positioning, MF exposure and increased chondrogenesis in ADSC. Chondrogenic induction of WJMSC was previously shown to result in increased upregulation of cartilage-specific type II collagen synthesis compared to BM-ADSC and decreased osteoinductive Runx2 and collagen X in similar culture conditions (Reppel et al., 2015), suggesting their use could avoid hypertrophic cartilage formation and graft ossification. Previous studies have used human BM ADSC loaded with homogenous sized MNP derived from bacteria (Magnetospirillium sp) for inducing cell aggregation in pellets and for delivering magnetic mediated shear stress during initial stages of chondrogenic conversion. Increased levels of GAGs and collagen production as well as the absence of hypertrophic conversion was observed in the MNP loaded cells (Son et al., 2015).

In our experience, MNP mediated aggregation in spheroid culture under MF was not efficient in increasing the amount of GAG production compared to simple pellet procedure, probably due to increase compaction of three dimensional structure and decreased nutrient availability (Labusca et al., 2021).

A limitation of this study is that we have not investigated hypertrophic markers. However, previous evidence supports the fact that significant GAG increase in ADSC-MNP at protein level is an indicator of normal articular cartilage, being correlated with collagen type expression II and absence of hypertrophy (Kamisan et al., 2013). Further studies will be needed as well to address donor variability especially for the case of ADSC. WJMSC donor population is more likely to being homogenous as it involves processing umbilical cord sample from women of fertile age and with healthy pregnancies. ADSC donors, however, could be more heterogeneous, especially if the use of autologous cells is envisaged for a future therapeutic purpose. A comparative study including a larger number of donors stratified based on age, gender and BMI as well as profiling ADSC characteristics relative to their chondrogenic conversion capability will to enable further donor selection for cartilage regeneration purposes.

## Conclusion

ADSC upload proprietary MNP by simple addition in culture media after 24 h in quantities that endows the cells with magnetic properties. ADSC-MNP, submitted to low-frequency MF during initial stages of chondrogenic induction, generated the highest amount of chondrogenic conversion at protein level as assessed by GAG/cell deposition after 21 days pellet culture. WJSC displayed low MNP internalization and significant lower amount of GAG deposition in similar conditions. B-Gal activity, after extended culture *in vitro*, was significantly decreased in ADSC-MNP compared to non-loaded, but not in WJMSC, suggesting a possible senescent protective mechanism that needs further investigation. Magneto-mechanical stimulation of MNP-loaded cells could be an efficient modality to increase the efficiency of ADSC, but not of WJMSC for cartilage engineering. Further studies are necessary to confirm the feasibility of this method in animal models of cartilage defects and to detect the molecular basis of MNP mediated magneto-mechanical stimulation.

## Data Availability

The raw data supporting the conclusion of this article will be made available by the authors, without undue reservation.
