# Supplementary material for: Magnetic Nanoparticles and Magnetic Field Exposure Enhances Chondrogenesis of Human Adipose Derived Mesenchymal Stem Cells But Not of Wharton Jelly Mesenchymal Stem Cells
Source: Front Bioeng Biotechnol. 2021 Oct 18;9:737132. doi: 10.3389/fbioe.2021.737132 (PMC8558412; doi:10.3389/fbioe.2021.737132)

Assessment of stem cell phenotype (accordingly to Dominici et al 2006)

1. All cells were cultured in 2D except chondrogenic pellets and were proved to be adherent to the polystyrene of tissue culture dishes.
2. Surface markers assessment was performed using a Guava flow cytometer (Guava Easycyte, Luminex Corporation) for thawed cells in P3, P4 or P5. Antibodies FITC/PE antibodies for CD105 (Invitrogen) CD 73 and 90 (BD Biosciences), were used as positive surface markers for stemness; CD 34 CD 45 (BD biosciences), negative surface markers, HLA-Dr (Invitrogen), γ1, γ2a (BD Biosciences) , negative controls and γ12b (BD Biosciences) as isotype control

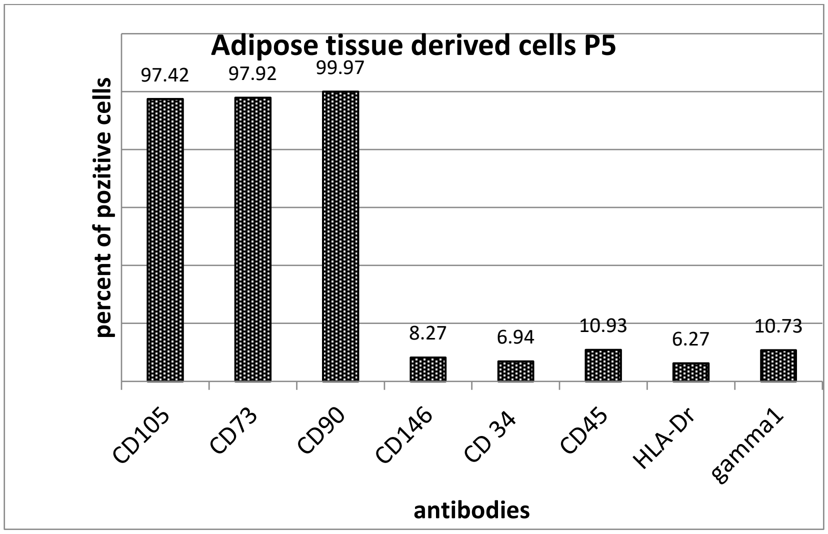

Supplm. chart 1. Percent of positive adipose derived cells for respective cluster of differentiation (CD) antigens within the gated population. Representative results from one donor

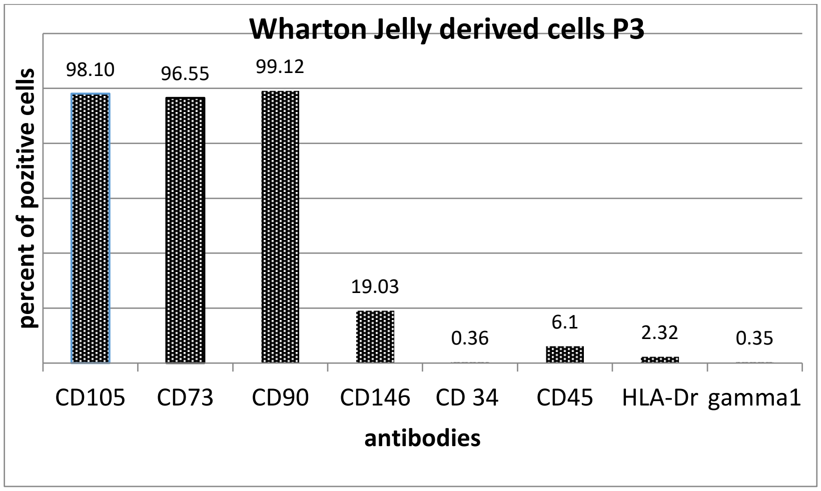

Supplm. chart 2. Percent of positive wharton jelly explanted cells for respective cluster of differentiation (CD) antigens within the gated population. Representative results from one donor

Minimal requirement for tri mesenchymal lineage differentiation

MNP loaded and non-loaded cells underwent trilineage differentiation (osteogenesis, adipogenesis, chondrogenesis- as presented in the main text of the manuscript)

Methods

Setup of osteogenesis and adipogenesis assays Cells in passage 2-4 were plated in 96 well plates at $1x10^4$cells/well, incubated until 95% confluent. MNPs were added to cells after suspending them in CCM or cells were left untreated with MNPs After 24 hours CCM was washed with PBS and replaced with osteogenetic (single Quots™ osteogenetic media Lonza) or with adipogenetic (single Quots™ adipogenetic induction and maintenance media Lonza) for osteogenesis or adipogenesis assays respectively, fed every each 3 days for 21 days (osteogenesis) or until completing three cycles of induction-maintenance for adipogenesis respectively. Cells were fixed with 98% ethanol for 10 minutes washed with PBS.

Assessment of differentiation – osteogenesis Osteoimage™ osteogenesis assay (Lonza, Walkersville US) was used for qualitative and quantitative assessment of osteogenesis, accordingly to provider's instructions; briefly fixed cells were washed with diluted Wash buffer (WB. 0, 5 ml /well of diluted staining reagent was added and plate incubated for 30 minutes at room temperature (RT), washed x3 with WB observed with inverted fluorescent microscope (Evos FL LifeTechnologies). For quantitative osteogenesis assay fluorescence was read at 492/520 excitation/emission using a plate reader.

Assessment of differentiation – adipogenesis Adipogenesis was quantified using AdipoRed™ assay (Lonza, Walkersville US) accordingly to provider's instructions, briefly cells were double washed with PBS and AdipoRed stain was added to culture wells. After a short incubation fluorescence was read in a plate reader at 485nm excitation and 524nm emission.

Adipogenesis

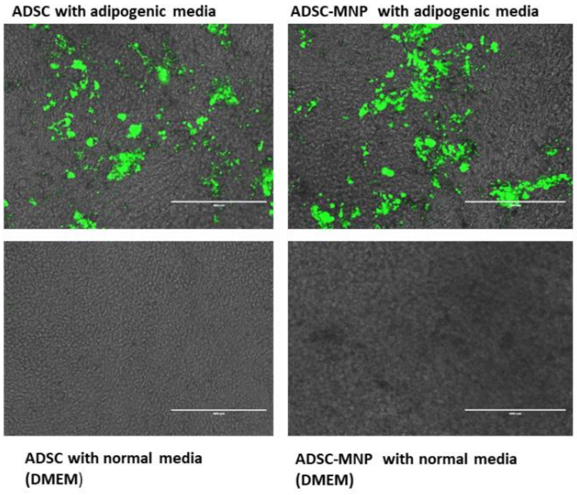

Osteogenesis

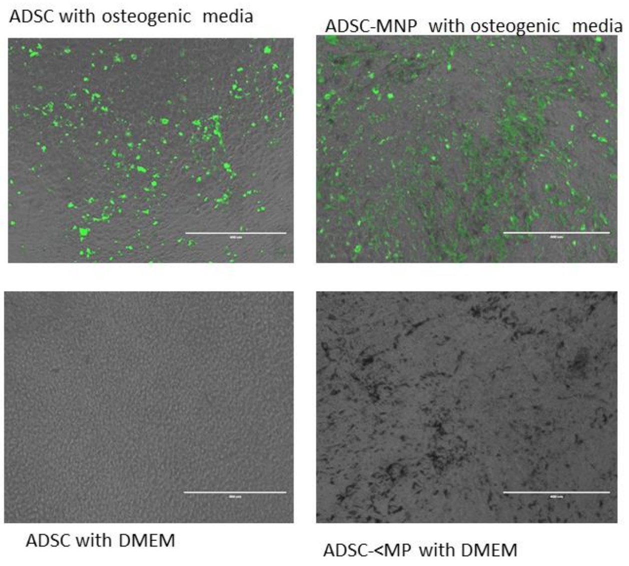

Supplement: Supplementary file 1 [file DataSheet1.PDF]
